# Draft Genome Sequence of *Bhargavaea cecembensis* Strain DSE10^T^, Isolated from a Deep-Sea Sediment Sample Collected at a Depth of 5,904 m from the Chagos-Laccadive Ridge System in the Indian Ocean

**DOI:** 10.1128/genomeA.00346-13

**Published:** 2013-06-13

**Authors:** Sisinthy Shivaji, Sreenivas Ara, Zareena Begum, Manorama Ruth, Aditya Singh, Anil Kumar Pinnaka

**Affiliations:** CSIR-Centre for Cellular and Molecular Biology, Habsiguda, Hyderabad, Andhra Pradesh, Indiaa; CSIR-Institute of Microbial Technology, Chandigarh, Indiab

## Abstract

Here, we report the 3.2-Mbp draft genome sequence of *Bhargavaea cecembensis* strain DSE10^T^, isolated from a sediment sample collected from the Chagos-Laccadive ridge system in the Indian Ocean at a depth of 5,904 m.

## GENOME ANNOUNCEMENT

About 100 novel species of bacteria have been isolated and identified worldwide in different deep-sea microbial projects. These studies have added 28 new genera to different bacterial families (1). Whole-genome sequencing of these deep-sea bacteria is likely to provide a better insight into the adaptation of these bacteria. In addition, it may provide additional information into novel pathways or enzymes that connect these unique bacteria with other deep-sea life forms and thus provide an evolutionary connection. *Bhargavaea cecembensis* strain DSE10^T^ was isolated from a deep-sea sediment sample from the Chagos-Laccadive ridge system in the Indian Ocean. The nearest phylogenetic neighbors of *B. cecembensis* strain DSE10^T^, according to the 16S rRNA gene analysis, are *Bhargavaea beijingensis* GE10 (99.48%), *Bhargavaea ginsengi* QE14^T^ (99.34%), *Chungangia koreensis* CAU 9163^T^ (96.43%), *Bacillus ginsengisoli* DCY53^T^ (96.12%), and *Bacillus fumarioli* LMG 17489^T^ (95.76%).

We sequenced the genome of *B. cecembensis* strain DSE10^T^ using the Roche 454 (FLX Titanium) pyrosequencing platform. The sequencing yielded 122,522,763 bases in 318,955 sequences, which is 38× coverage of the genome. The sequence was assembled using GS *de novo* Assembler (version 2.8; F. Hoffmann-La Roche Ltd., Switzerland). Assembly yielded 39 contigs, all of which were ≥555 bp, with the largest contig being 374,540 bp long and an average contig length of 82,242 bp. All contigs were above the quality score of 40, with an average quality score of 63.87. The G+C mol% of *B. cecembensis* strain DSE10^T^ was found to be 54.83%. The annotation of the genome was done using Prokaryotic Genome Annotation System (PROKKA) pipeline (2), tRNA was predicted by tRNAscan-SE 1.23 (3), and rRNA genes were predicted by RNAmmer 1.2 (4). The draft genome of *B. cecembensis* strain DSE10^T^ consists of 39 contigs, for a total length of 3,207,463 bp.

The annotation has 3,288 genes, including 63 tRNA and 6 rRNA genes. Out of the 3,288 coding regions, 3,169 (96.38%) were functionally annotated. The annotated genome has 108 genes for membrane transport, including 37 genes for ABC transporters. Thirty-eight genes encode resistance against antibiotics, and 8 genes are for the bacitracin stress response. The annotation also reports the presence of one *Listeria* pathogenicity island, LIPI-1. There were 86 genes for bacterial stress response, including 16 heat shock, 2 cold shock, and 13 detoxification genes. There are also 4 genes for auxin biosynthesis.

Functional comparison was done using the SEED Framework for Comparative Genomics (http://www.theseed.org). It revealed *Bacillus* sp. B14905, *Bacillus* B-14905, *Lysinibacillus sphaericus* C3-41, *Bacillus licheniformis* ATCC 14580, *B. licheniformis* ATCC 14580, and *Anoxybacillus flavithermus* WK1 to be the closest neighbors of *B. cecembensis* DSE10^T^, with similarity scores of 513, 473, 465, 315, 306, and 282, respectively.

The sequencing of the genome will assist in understanding the evolution and microbial diversity of the deep-sea habitat.

### Nucleotide sequence accession number.

The draft genome sequence of *B. cecembensis* DSE10^T^ has been deposited in DDBJ/EMBL/GenBank under the accession no. AOFT00000000. The version described in this paper is the first version.
